# The Emerging Roles of Multimolecular G-Quadruplexes in Transcriptional Regulation and Chromatin Organization

**DOI:** 10.1021/acs.accounts.4c00574

**Published:** 2024-11-18

**Authors:** Naura Fakhira Antariksa, Marco Di Antonio

**Affiliations:** † Imperial College London, Department of Chemistry, Molecular Sciences Research Hub, 82 Wood Lane, London W12 0BZ, U.K.; ‡ The Francis Crick Institute, 1 Midland Road, London NW1 1AT, U.K.; § Institute of Chemical Biology, Molecular Sciences Research Hub, 82 Wood Lane, London W12 0BZ, U.K.

## Abstract

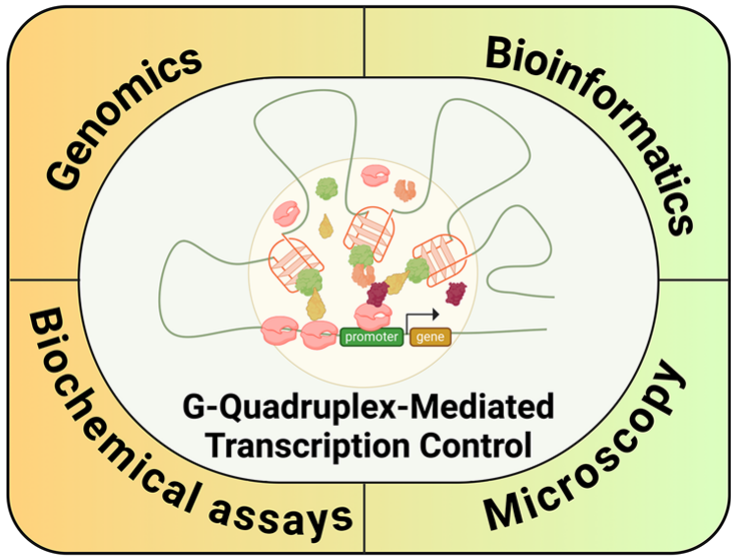

The ability of genomic DNA to adopt non-canonical secondary structures known as G-quadruplexes (G4s) under physiological conditions has been recognized for its potential regulatory function of various biological processes. Among those, transcription has recently emerged as a key process that can be heavily affected by G4 formation, particularly when these structures form at gene promoters. While the presence of G4s within gene promoters has been traditionally associated with transcriptional inhibition, in a model whereby G4s act as roadblocks to polymerase elongation, recent genomics experiments have revealed that the regulatory role of G4s in transcription is more complex than initially anticipated. Indeed, earlier studies linking G4-formation and transcription mainly relied on small-molecule ligands to stabilize and promote G4s, which might lead to disruption of protein–DNA interactions and local environments and, therefore, does not necessarily reflect the endogenous function of G4s at gene promoters. There is now strong evidence pointing toward G4s being associated with transcriptional enhancement, rather than repression, through multifaceted mechanisms such as recruitment of key transcriptional proteins, molding of chromatin architecture, and mode of phase separation.

In this Account, we explore pivotal findings from our research on a particular subset of G4s, namely, those formed through interactions between distant genomic locations or independent nucleic acid strands, referred to as multimolecular G4s (mG4s), and discuss their active role in transcriptional regulation. We present our recent studies suggesting that the formation of mG4s may positively regulate transcription by inducing phase-separation and selectively recruiting chromatin-remodeling proteins. Our work highlighted how mG4-forming DNA and RNA sequences can lead to liquid–liquid phase separation (LLPS) in the absence of any protein. This discovery provided new insights into a potential mechanism by which mG4 can positively regulate active gene expression, namely, by establishing DNA networks based on distal guanine–guanine base pairing that creates liquid droplets at the interface of DNA loops. This is particularly relevant in light of the increasing evidence suggesting that G4 structures formed at enhancers can drive elevated expression of the associated genes. Given the complex three-dimensional nature of enhancers, our findings underscore how mG4 formation at enhancers would be particularly beneficial for promoting transcription. Moreover, we will elaborate on our recent discovery of a DNA repair and chromatin remodeling protein named Cockayne Syndrome B (CSB) that displays astonishing binding selectivity to mG4s over the more canonical unimolecular counterparts, suggesting another role of mG4s for molding chromatin architecture at DNA loops sites.

Altogether, the studies presented in this Account suggest that mG4 formation in a chromatin context could be a crucial yet underexplored structural feature for transcriptional regulation. Whether mG4s actively regulate transcription or are formed as a mere consequence of chromatin plasticity remains to be elucidated. Still, given the novel insights offered by our research and the potential for mG4s to be selectively targeted by chemical and biological probes, we anticipate that further studies into the fundamental biology regulated by these structures can provide unprecedented opportunities for the development of therapeutic agents aimed at targeting nucleic acids from a fresh perspective.

## Key References

- LianoD.; ChowdhuryS.; Di AntonioM.Cockayne Syndrome B Protein Selectively Resolves and Interact with Intermolecular DNA G-Quadruplex Structures. J. Am. Chem. Soc.2021, 143 ( (49), ), 20988–2100234855372 10.1021/jacs.1c10745.^1^*This work is the first to report a human protein that displays high specificity to multimolecular G4s, hinting at the potential biological significance of these structures in the context of transcriptional regulation*.
- RaguseoF.; WangY.; LiJ.; Petrić HoweM.; BalendraR.; HuyghebaertA.; VadukulD. M.; TanaseD. A.; MaherT. E.; MaloufL.; Rubio-SánchezR.; AprileF. A.; ElaniY.; PataniR.; Di MicheleL.; Di AntonioM.The ALS/FTD-Related C9orf72 Hexanucleotide Repeat Expansion Forms RNA Condensates through Multimolecular G-Quadruplexes. Nat. Commun.2023, 14 ( (1), ), 827238092738 10.1038/s41467-023-43872-1PMC10719400.^2^*This work demonstrated that complex matrixes mediated by formation of multimolecular G4s can lead to phase separation. This can be relevant in the context of neurodegeneration and formation of pathological aggregates and, potentially, in the context of transcriptional regulation*.
- RobinsonJ.; FlintG.; GarnerI.; GalliS.; MaherT. E.; KuimovaM. K.; VilarR.; McneishI. A.; BrownR.; KeunH.; AntonioM. Di.G-Quadruplex Structures Regulate Long-Range Transcriptional Reprogramming to Promote Drug Resistance in Ovarian Cancer. bioRxiv202410.1186/s13059-025-03654-yPMC1225511640646632, 10.1101/2024.06.24.600010.^3^*This work demonstrated the importance of nonpromoter G4s in controlling gene expression. Our integrated genomics and bioinformatics analyses laid the foundation for a novel concept of G4-mediated transcriptional regulation via long-range DNA interactions through the formation of G4-clusters*.

## Introduction

### G-Quadruplexes (G4s) are Relevant in a Variety of Biological Contexts

**Figure 1:**
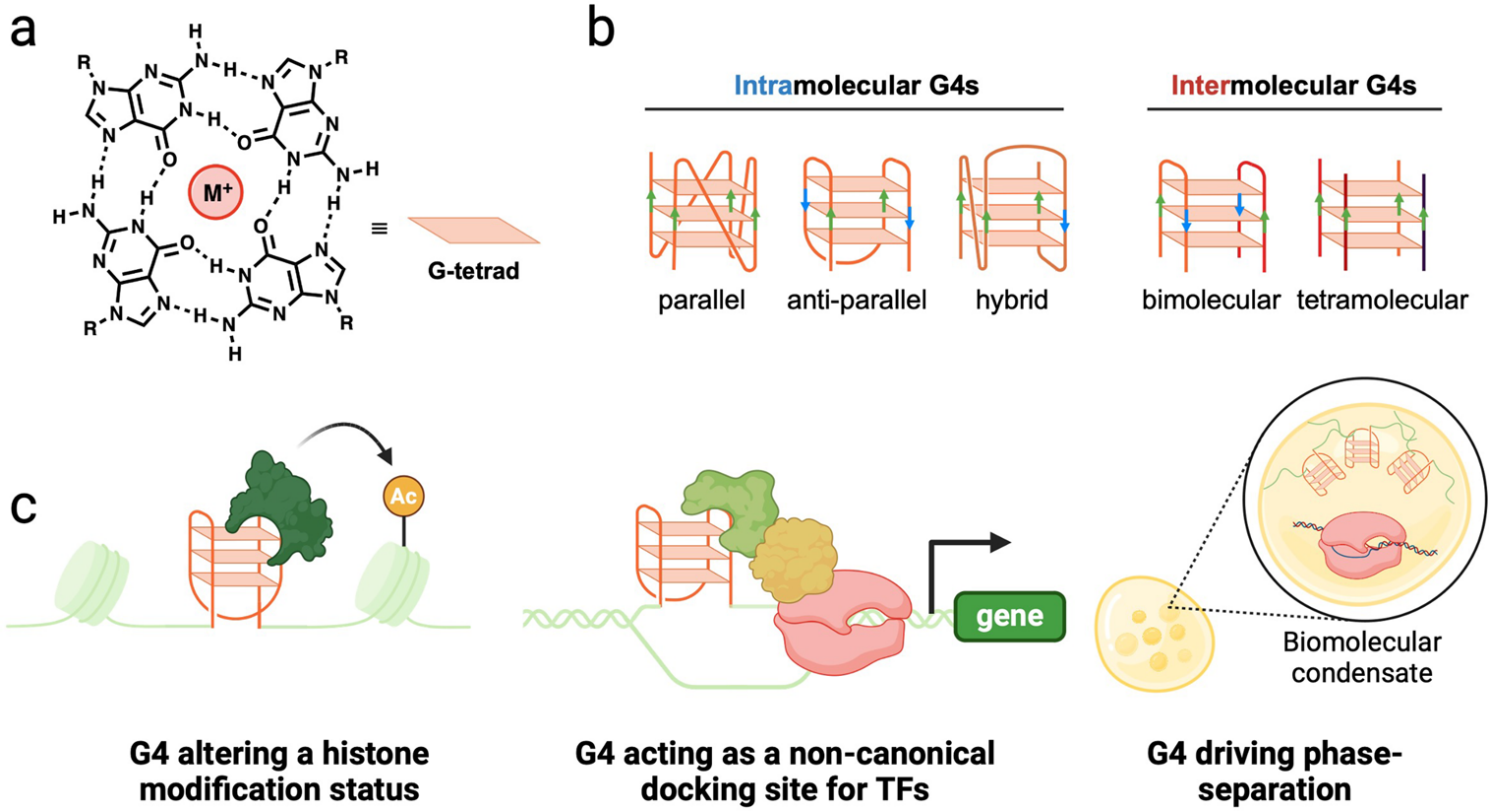
General structural features of G-quadruplexes and their biological relevance. (a) Structure of a G-tetrad composed of four guanines interacting by Hoogsteen hydrogen bonding. M^+^ refers to a monovalent cation, with the order of stability K^+^ > Na^+^ > Li^+^. (b) Schematic representation of different G4 topologies and molecularities. Bimolecular and tetramolecular G4s are shown on the right, whereas different topologies of monomolecular G4s are displayed on the left. (c) Schematic illustration of the various biological processes by which G4 structures have been postulated to play a role in transcriptional regulation.

Guanine-rich nucleic acid sequences can fold into noncanonical, alternative secondary structures known as G-quadruplexes (G4s). These structures are formed through the mutual interaction of four guanines via Hoogsteen hydrogen bonding, resulting in a planar arrangement called the G-tetrad (Figure 1a). The stacking of two or more G-tetrads generates a complete G-quadruplex structure (Figure 1b), which is stabilized by the coordination of a monovalent cation to the O-6 lone pair electrons (with the stability order being K^+^ > Na^+^ > Li^+^). The consensus sequence of G_3–5_N_1–7_G_3–5_N_1–7_G_3–5_N_1–7_G_3–5_ is often utilized in bioinformatic algorithms to identify putative G4-structures within the genome.^4,5^ Here, the four G_3–5_ repeats represent the so-called “G-tracts” that interact by hydrogen bonding to form three to five stacked G-tetrads. These G-tracts are connected by three different sequences of any base composition between 1 and 7 nucleotides (N_1–7_), referred to as “loops”. Despite the relatively simple consensus sequence, G4 structures can be complex and exhibit significant polymorphism by adopting different folding topologies (Figure 1b).^6−8^ This polymorphism is influenced by differences in the sequence length and base composition of the loops, as well as from the relative nucleic acid strand orientation in the context of the formed G4 (Figure 1b). Additionally, the stoichiometry of the guanine-rich strands affects the molecularity of the assembled G4, allowing either unimolecular (formed from a single nucleic acid strand, i.e. *intra*molecular) or multimolecular (formed from two to four nucleic acid strands, i.e. *inter*molecular) G4s to form (Figure 1b).

Over the past two decades, a combination of chemical biology, bioinformatics, and genomic approaches has uncovered the widespread presence of G4s in various functional regions of the human genome. Putative G4 forming sequences are abundant in the telomeric region,^9^ gene-promoters,^10,11^ 3′ and 5′ untranslated regions, origins of replication,^12^ etc., alluding to their potential for modulating various biological processes. Among those, G4s in promoters are of particular interest due to their potential as drug targets for interfering with the expression of undruggable genes.^13^ Early research in this area revealed that the promoters of oncogenes, such as MYC, KRAS, and c-KIT, contain G4 structures.^14,15^ Targeting these G4s with G4-binding ligands has been shown to downregulate the expression of these oncogenes. Gene downregulation elicited by G4-ligands has often been rationalized by a roadblock effect that the stabilized G4 structure can have on transcriptional polymerases. However, there is now overwhelming evidence indicating that promoter G4s may display a more intricate interplay with proteins and chromatin architecture, which leads to active transcription rather than its repression.

### Challenging the Transcriptional-Repressor Model

Computational predictions indicated that about 10,000 human genes contain at least one putative G4-forming sequence within 1 kb upstream of the transcription start sites.^10,11^ Formation of G4s within these promoters was traditionally linked to transcriptional repression, as they were thought to present a blockage that impedes the elongation of RNA polymerase (RNAP), thereby stalling transcription.^11,16,17^ However, it is important to note that these early studies were conducted in an *in vitro* context, typically using model plasmids or linear oligonucleotides, which do not accurately recapitulate the endogenous transcription environments in living cells. More importantly, most of these studies relied on using small-molecule ligands to stabilize G4s, which has been later shown to induce DNA damage and transcriptional stalling when the ligands are bound to G4s.^18^ Moreover, small molecule ligands may also affect the folding dynamics of G4s and their binding to endogenous proteins.^19,20^ It has now become apparent that the biological role of endogenous G4s might not reflect what is observed through the ligand-bound structures. These issues highlighted the importance of investigating G4s within the native chromatin environment to pinpoint their exact functional role in transcriptional regulation. Indeed, subsequent genomics studies have revealed that G4s in gene promoters of various cell lines and tissue models are associated with actively transcribed genes rather than downregulated ones as initially postulated.^21,22^ Single-molecule imaging of G4s has also shown that the formation of this structure is dynamic and linked to active transcription in the cell cycle.^23^ These findings suggested a new paradigm in which G4s play a more complex role in transcription beyond merely acting as a blockage to RNAPs.

Recent literature has provided compelling evidence demonstrating that G4s can upregulate gene expression through various mechanisms (Figure 1c). For example, unresolved G4s accumulated during DNA replication induce the loss of the histone modification H3K9 dimethylation (an inactive transcription marker) and the incorporation of acetylated histones (an active transcription marker) around the G4 site.^24^ These changes in the epigenetic status eventually led to the upregulation of the p-globin locus. Another mechanism by which G4s elevate transcription is by serving as non-canonical docking sites for transcription factors (TFs).^25^ It has been shown that the same G4 structure can promiscuously bind various TFs both *in vitro* and in a cellular context. This suggests that endogenous G4s could act as hubs for the engagement of multiple TF complexes, resulting in more frequent recruitment of RNAP II and, consequently, increased transcription. Interestingly, multiple G4s have also been shown to trigger a phase separation event by forming an interconnected network of nucleic acid strands, which might lead to transcriptional enhancement due to changes in the local environment rather than directly binding to regulatory factors.^26,27^ Burrows and co-workers have even shown that DNA damage leading to G4-formation can recruit DNA-repair complexes that promote gene expression at G4-forming sites,^28^ clearly highlighting how diverse and context-dependent the biological response elicited by G4-formation can be. More recently, the Balasubramanian group has demonstrated that inserting a G4-forming sequence taken from the KRAS promoter region into the MYC promoter using CRISPR-Cas9 technologies would also lead to increased MYC expression.^29^ This paradigm-shifting work further demonstrates how the structural feature of a G4 within a promoter, rather than its sequence, can stimulate transcriptional regulation.

Our group and others have also associated G4-formation with driving long-range DNA interactions, providing yet another mechanism by which these structures may influence transcription. Indeed, G4s are abundant at DNA loop boundaries, suggesting a role akin to the CTCF protein in stalling the cohesin complex and stabilizing the DNA loop.^30,31^ This loop stabilization may bring promoters and distal regulatory elements, such as enhancers, into proximity, allowing control of gene expression over long genomic distances. Furthermore, G4s may further stabilize the DNA loop by directly binding regulatory proteins, including those involved in transcriptional activation, such as BRD3^32^ and YY1.^33^ These findings highlight a fascinating interplay among long-range DNA interactions, gene regulation, and G4 formation, which we anticipate can be transformative in developing therapeutic agents that target G4s.

Our group has recently generated compelling evidence indicating that long-range G4 interactions could drive transcriptional enhancement and that the formation of multimolecular G4s (mG4s) can be biologically significant.^3,8^ In this Account, we will discuss the potential relevance of mG4 formation at nonpromoter regulatory regions, such as enhancers, and elaborate on our investigation into phase separation events mediated by highly G-rich sequences. Phase separation may play a critical role in the transcriptional enhancement observed in G4-containing enhancers and superenhancers. Finally, we present our findings on a chromatin remodeling protein that can selectively recognize multimolecular G4s over unimolecular ones, which further highlights the potential biological relevance of multimolecular G4s and their association with chromatin architecture and transcriptional regulation.

## The Role of Non-promoter G4s in Transcriptional Regulation

Most of the literature linking G4-formation to transcriptional regulation has focused on promoters, given the high abundance of these structures at gene promoters. Moreover, genomic studies have also revealed that promoter G4s are associated with the highest changes in gene expression detected by RNA-Seq, further confirming a key role of G4-formation at gene promoters.^18^ However, recent evidence also indicates that G4 structures formed at intergenic regions, such as enhancers, can also stimulate transcription. Indeed, the Borchert group has suggested that long-range G4s can promote enhancer-like structures, bringing gene promoters in proximity of transcriptional activators.^34^ Moreover, they have computationally predicted that such long-range G4s are particularly enriched at established enhancer sites, hinting at a potential cooperative effect between G4s and enhancers.^34^ Similarly, artificially inserting highly G-rich sequences that can form an array of G4s using CRISPR-Cas9 within active gene promoters has revealed that G4 structures can facilitate the establishment of novel long-range chromatin interactions, stimulating transcription in a similar way to what is observed with canonical enhancers.^35^ This strongly indicates that clusters of G4s can potentially mold the chromatin architecture in their own right and establish higher-order structures reminiscent of enhancers but driven by G4-based interactions.

**Figure 2:**
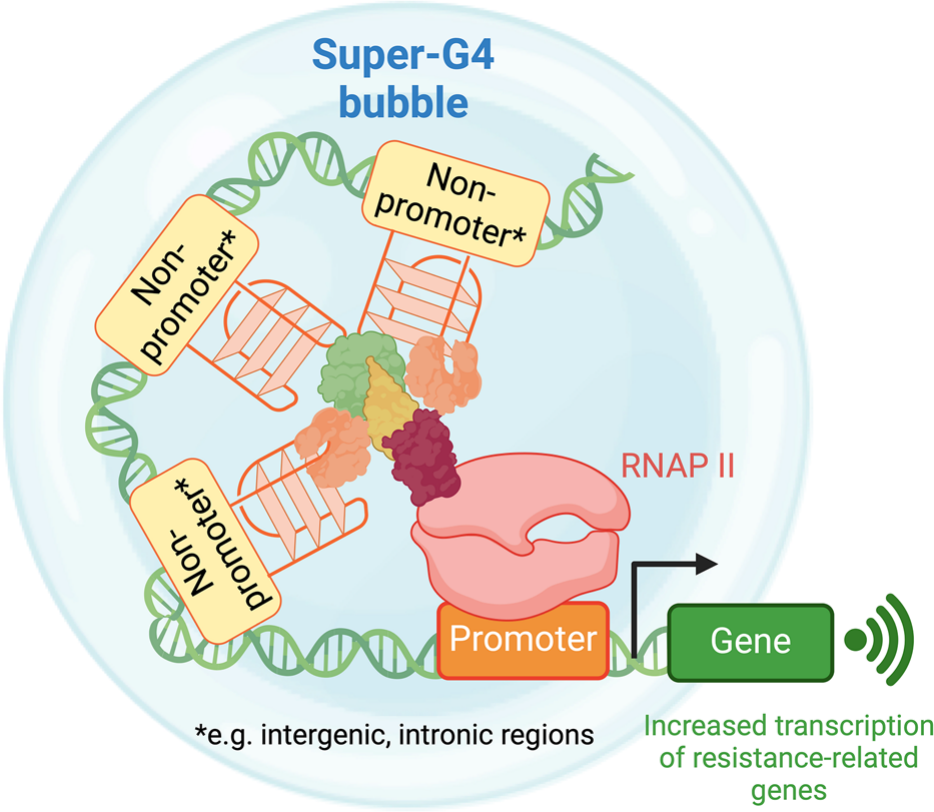
Proposed formation mechanism of a “super-G4” cluster by nonpromoter G4s. Super-G4 leads to epigenetic rewiring correlated to the increasing expression of genes important for conferring drug resistance in ovarian cancer. Figures were reproduced from ref (3). Available under a CC-BY ND license. Copyright 2023 Robinson et al.

In a recent study in our group, we have also confirmed the enrichment of G4s at enhancers and superenhancers in a chemoresistant ovarian cancer cell model (PEO4), indicating that G4-containing enhancers represent a subcategory of particularly potent transcriptional activating sites.^3^ Moreover, we found that in PEO4 cells promoter G4s had only a modest effect in stimulating transcription. In contrast, enhancer- and superenhancer-linked G4s demonstrated a particularly potent ability to elevate the transcription of genes linked to the acquisition of chemoresistance. These observations allowed us to propose a model by which G4s at intergenic and intronic regions, rather than promoters, were the key driver of drug resistance in ovarian cancer, which is in agreement with the recent literature suggesting a role of G4-formation at enhancer sites (Figure 2).

Moreover, we also questioned whether clusters of G4s could act cooperatively with each other and stimulate further transcriptional activation, similarly to what was observed for clusters of enhancers (i.e., superenhancers). This hypothesis was substantiated by the fact that Chowdhury and co-workers reported that an array of G4s, rather than an individual structure, is required to establish novel long-range interactions.^35^ Our study revealed that a cluster of G4s, which we termed “super-G4” in a reminiscent way of superenhancers, exhibited significantly elevated gene expression, surpassing the increase of expression levels associated with regular superenhancers, suggesting that super-G4s might represent an independent epigenetic feature to regulate gene expression. We also anticipate that given the high G4-density in super-G4s, it is likely that these structures could lead to formation of multimolecular G4s, thus representing a potential novel therapeutic target for epigenetically rewiring ovarian cancer cells to reverse drug resistance.

Based on recent literature and our studies, it is increasingly evident that the transcriptional regulation mediated by G4s is not limited to promoters. Clusters of G4s, either endogenous or artificially inserted, are linked with the establishment of long-range chromatin interactions, allowing them to behave like superenhancers and stimulate transcriptional activation to a greater extent than what is measured for single G4-formation at promoters (Figure 2). The exact mechanism by which these G4-clusters achieve this superenhancer ability remains to be elucidated. Still, it is plausible that these sites may act as hubs for regulatory proteins or trigger phase separation events, leading to elevated transcription. Nevertheless, these observations offer an exciting new perspective on G4-mediated gene regulation that goes beyond individual gene promoters, highlighting the potential for druggability of super-G4s for therapeutic purposes.

Given the unique chemical and structural features characterizing multimolecular G4 structures, this motif can likely be exploited in many other mechanisms that regulate chromatin architecture and epigenetic regulation. The fast development of orthogonal genomics strategies to map G4s and other epigenetic marks will greatly facilitate the discovery of such pathways, offering a great opportunity for the chemical community to develop novel ligands to interfere with such processes.

### G-Rich Intron Sequences Can Form a Multimolecular-G4 That Phase Separates

Our data and current literature indicate that clusters of G4s (i.e., super-G4s) can act as hubs for transcriptional enhancement. However, the precise mechanism behind the formation of these G4-clusters and their role in promoting gene expression remain to be elucidated. One possible mechanism heavily linked to the formation of superenhancers and transcription factors is the stimulation of liquid–liquid phase separation (LLPS). This phenomenon leads to the formation of liquid droplets capable of sequestering and concentrating transcriptional regulatory proteins. In superenhancers, LLPS involves weak interactions between nucleic acids and regulatory proteins, which increase the local concentration of these proteins, thereby elevating transcription.^36^ Therefore, it is conceivable that LLPS might also be relevant for G4 clusters, where the formation of G4-based matrixes can stimulate condensation. In this section, we elaborate on a key finding from our group that demonstrates how G-rich sequences can indeed lead to LLPS events in a protein-independent manner, a concept that could be readily applicable to the context of transcriptional regulation.

**Figure 3:**
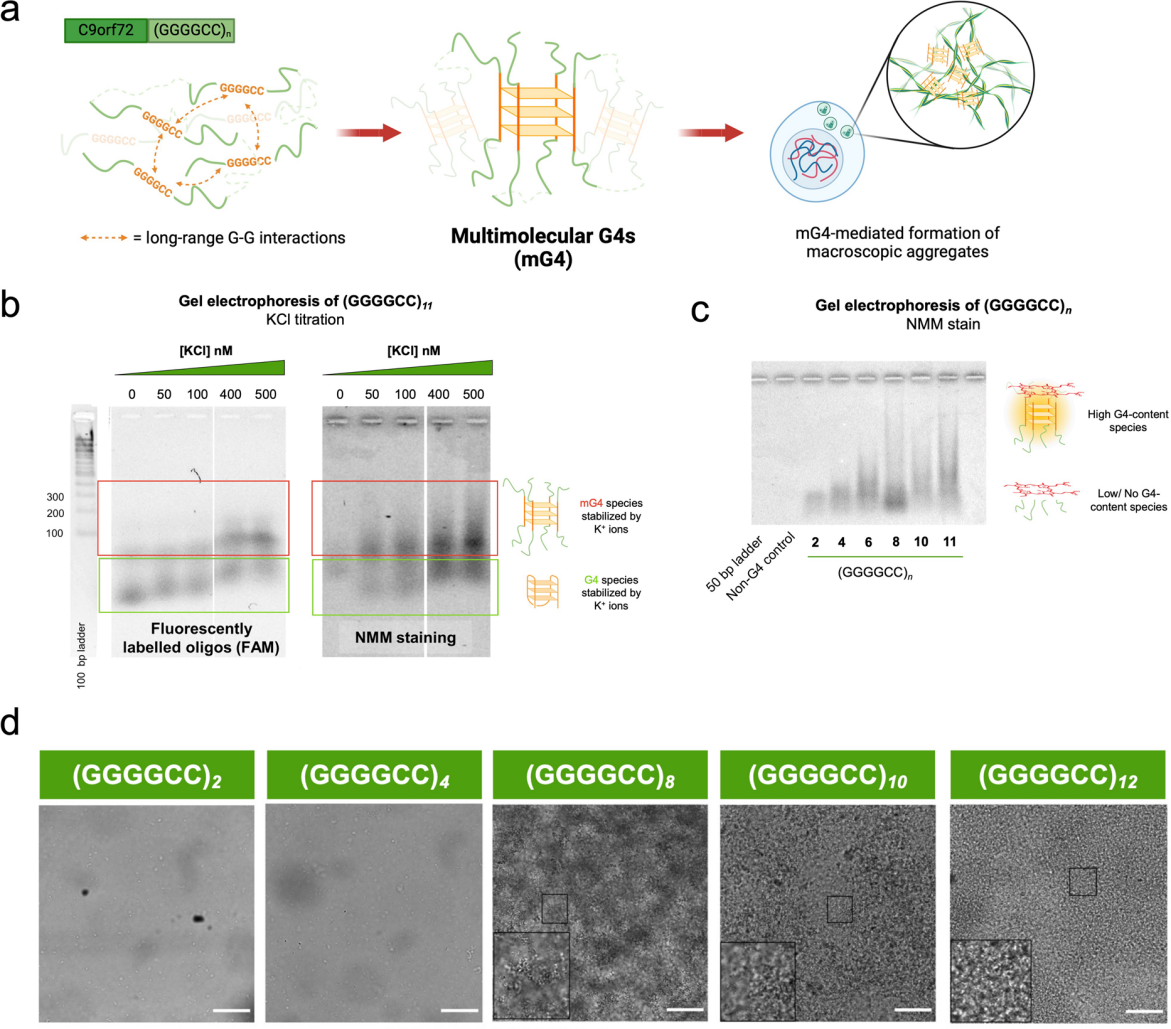
Multimolecular G4s cluster and form a phase-separated entity in the (GGGGCC)_*n*_ hexanucleotide repeat. (a) Proposed mechanism of G4-mediated aggregation: (GGGGCC)_*n*_, by virtue of being G-rich, forms multimolecular G4s (mG4), which further cluster into microscopic aggregates. (b) Fluorescence (left) and NMM (right) gels demonstrate the formation of mG4 species, indicated by the appearance of a higher molecular weight species with increasing concentration of K^+^ (G4 stabilizing cation). (c) NMM gel shows that mG4 formation depends on the number of (GGGGCC)_*n*_ repeats; a higher number of repeats allows better cross-linking between strands, which more readily form aggregates. (d) Bright field imaging of (GGGGCC)_*n*_ (*n* = 2–12) annealed under mG4-forming conditions at 250 μM oligonucleotide concentration. 100 μm scale bar. Figures were reproduced from ref (2). Available under a Creative Commons CC BY license. Copyright 2023 Raguseo et al.

In the neurodegenerative diseases amyotrophic lateral sclerosis (ALS) and frontotemporal dementia (FTD), the expansion of the intronic hexanucleotide repeat (GGGGCC)_*n*_ in the *C9orf72* locus is the most common hereditary tract. Given the guanine-rich nature of this sequence, it has been shown to form G-quadruplex structures in both its DNA and RNA forms.^37^ At the RNA level, this sequence is also known to form aggregates *in vitro* upon reaching a critical number of repeats,^38^ potentially serving as the nucleation site for phase-separated aggregates to form, which are typical of neurodegenerative diseases. Our study expanded on this notion by demonstrating the mechanism by which (GGGGCC)_*n*_ repeats aggregate, specifically by forming three-dimensional interconnected linkages via long-range G–G interactions, resulting in what are known as multimolecular G-quadruplexes (mG4s) (Figure 3a).

Initially, we performed a simple agarose gel electrophoresis experiment on the DNA (GGGGCC)_*n*_ sequence, which was annealed under mG4-forming conditions (K^+^ containing buffer, crowding conditions, and slow annealing time). This experiment revealed the presence of two G4 species, as detected by in-gel fluorescence and G4-specific *N*-methyl mesoporphyrin IX (NMM) staining,^39,40^ suggesting that the slower migrating band represents the mG4 (Figure 3b). To confirm this, we observed that decreasing the concentration of K^+^ in the buffer or decreasing the number of repeats (Figure 3c) destabilized the slow-running species, confirming that the slow-running band could indeed be ascribed to mG4 structures.

Having confirmed the propensity of the DNA (GGGGCC)_*n*_ sequence to form mG4s, we demonstrated via confocal microscopy that the formed mG4s could indeed lead to the formation of macroscopic aggregates (Figure 3d). We also showed that this phenomenon occurs for the (GGGGCC)_*n*_ sequence in its RNA form, even at lower oligonucleotide concentrations than its DNA counterpart. In this *in vitro* study, we observed that the mG4 aggregates display a solid- or gel-like morphology. Nevertheless, it is possible that in a cellular environment, the mG4 aggregates may subsequently act as a protein docking site, leading to a more liquid-like biomolecular condensate often associated with ALS/FTD aggregates. Indeed, our further investigations showed that the presence of (GGGGCC)_*n*_ mG4-mediated aggregates enhanced the binding and aggregation of the RNA-processing protein heavily linked with ALS and FTD, TDP-43.

Additionally, we found that treatment with the G4-binding ligand pyridostatin (PDS) during the mG4 annealing process prevents the formation of macroscopic aggregates. PDS is known to stabilize unimolecular G4s over multimolecular ones.^20^ Our data demonstrated that such G4-stabilizing ligands perturb the folding dynamics of G4s by preferentially stabilizing a specific G4 subtype, thereby affecting the G4s’ ability to undergo phase separation. Interestingly, we similarly observed significant transcriptional down-regulation at super-G4 sites induced by PDS treatment in ovarian cancer cells.^3^ This might suggest that the collapse of mG4-mediated biomolecular condensates at super-G4 sites upon PDS treatment might be responsible for the observed strong ligand-induced downregulation of genes under the control of super-G4s.^3^

While the (GGGGCC)_*n*_ repeat expansion study was conducted within the context of neurodegeneration, the fact that mG4s drive the formation of biomolecular condensates could potentially be applied to explain the beneficial role of high G4-density in enhancers and superenhancers. In this scenario, individual G4s from distal strands of genomic DNA could come together through chromatin looping, creating a local structural hub for interactions with transcriptional regulatory proteins. This would generate the crowding conditions necessary for LLPS, as demonstrated in the mG4–TDP-43 aggregate formation.^37^ Consequently, it is conceivable that such a phase-separated G4 site would increase the local concentration of regulatory proteins, leading to efficient enhancement of gene expression. We anticipate that the development of rigorous biophysical models to simulate super-G4 behavior *in vitro* must be developed to investigate this hypothesis further.

It is increasingly evident that mG4s might form promptly under physiological conditions and potentially at key regulatory regions. The increasing knowledge and generation of chemical biology tools to study DNA looping and LLPS will play a key role in defining whether mG4s can indeed form in a chromatin context and promote transcriptional regulation by LLPS or other mechanisms. In this context, developing novel genomic methods to capture mG4s formation in chromatin will be key to unequivocally addressing this question.

### A Chromatin Remodeler Protein That Selectively Binds to mG4s

It is known that one mechanism for achieving transcriptional activation involves the formation of biomolecular condensates that sequester transcriptional activating proteins. Indeed, our research suggested that long-range G4s (mG4s) have the potential to trigger phase separation states (biomolecular condensates) that could indeed be linked to enhanced transcription.

Transcriptional condensates typically comprise RNAP II, TFs, and transcriptional co-activators, such as the Mediator complex, which drives LLPS due to the intrinsically disordered nature of the protein rather than nucleic acids.^41−43^ Interestingly, emerging evidence indicates that chromatin remodeling proteins, besides transcriptional activators, are also recruited into these condensates.^44^ This demonstrates that condensate formation is also associated with increased chromatin accessibility, suggesting that condensates can regulate transcription not only by recruiting transcriptional activators but also by recruiting epigenetic remodeling proteins to alter the chromatin structure. It is thus conceivable that chromatin-remodeling proteins might also regulate the formation of higher-order nucleic structures, such as mG4, leading to both chromatin remodeling and LLPS.

Excitingly, we recently discovered that a chromatin-remodeling protein, Cockayne Syndrome B (CSB), displays a high affinity and selectivity for mG4s over more canonical unimolecular ones. This suggests that the CSB may potentially be involved in assembling or detecting mG4 networks in the context of chromatin remodeling. The protein is traditionally known for its role in transcription-coupled nucleotide excision repair, with its mutation leading to Cockayne syndrome (CS), a severe premature aging disease.^45,46^ Although the exact mechanism of CSB-mediated repair remains elusive, biochemical studies have revealed that CSB exhibits chromatin-remodeling activity, specifically by wrapping and unwrapping DNA strands in an ATP-dependent manner.^47,48^ These results suggest that CSB may alter the chromatin conformation to enhance accessibility for efficient DNA repair processes. Therefore, its ability to bind with high affinity and selectivity to mG4s might indicate that CSB can promote chromatin accessibility at mG4 sites.

**Figure 4:**
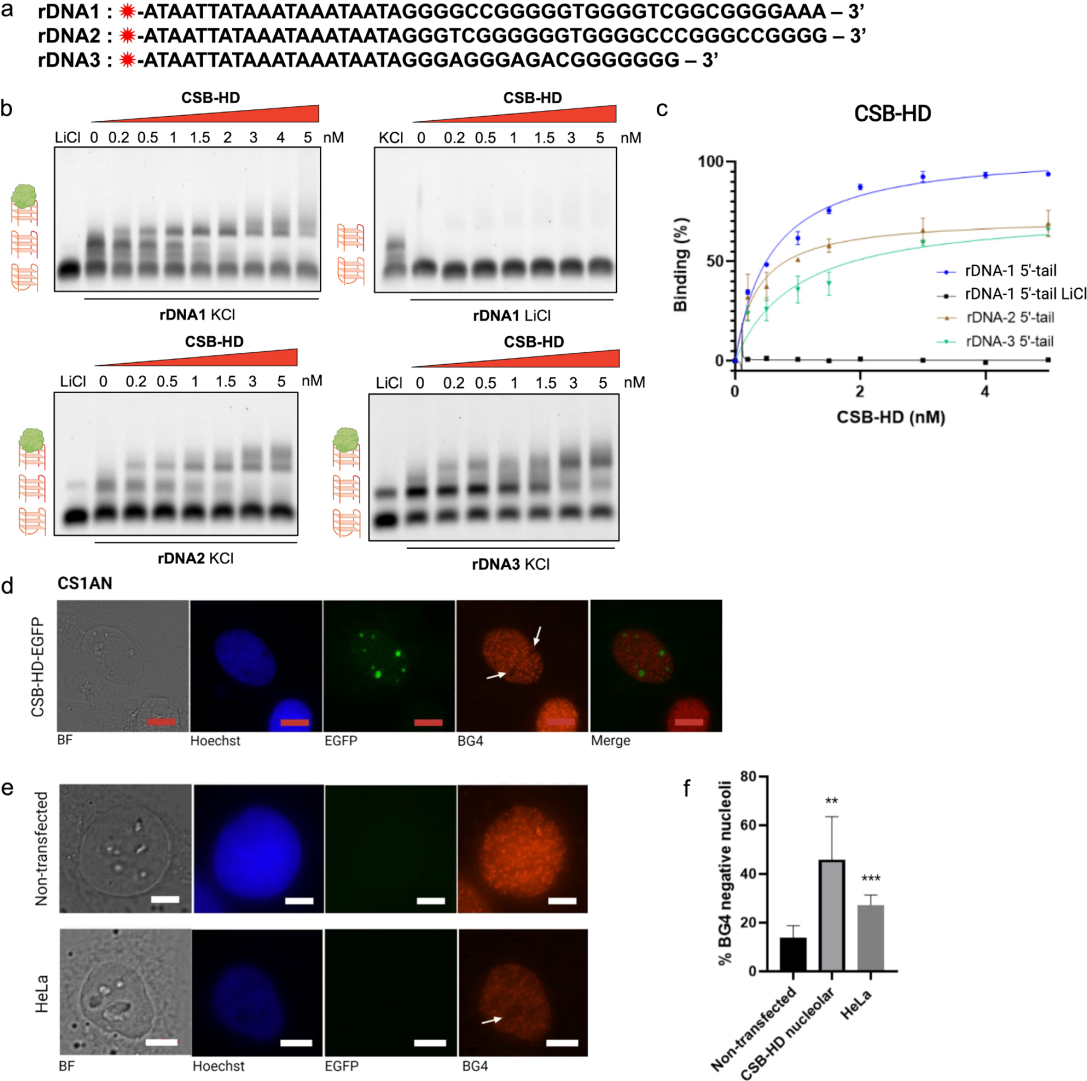
CSB binds multimolecular G4s (mG4s) with high affinity and specificity. (a) rDNA sequences utilized in the EMSA experiment. The red star symbol refers to the Cy5 dye. (b) EMSA gels on rDNA1 (annealed in KCl and LiCl), rDNA2, and rDNA3 G4s under 0–5 nM of CSB-HD. (c) Binding isotherm showing the percentage of CSB-HD bound intermolecular G4 under increasing protein concentration. The gel images were analyzed using ImageJ, and the binding affinity (*K*_D_) was calculated using Prism, fitting the binding curve to the “one site-specific binding” equation. All of the experiments were performed in biological duplicates. (d) Nucleoli localization of CSB protein in CSB-EGFP-expressing CS1AN cells. As the nuclei were occupied by CSB, probing with a G4-specific antibody, BG4, is inefficient, resulting in black spots in the nuclei locale. (e) Nucleoli of nontransfected CS1AN (top) and HeLa (bottom) cells, both of which are CSB-null, are efficiently stained by BG4. (f) Quantification of the number of cells *without* BG4 signal in nontransfected CS1AN cells, CSB-reinstated CS1AN cells, and HeLa cells. Figures were reproduced from ref (1). Copyright 2021 American Chemical Society.

The connection between CSB and G4s was initially highlighted by findings linking CS disease with aberrant ribosomal DNA (rDNA) transcription.^49^ Given the guanine-rich nature of rDNA, it readily forms G4 structures under physiological conditions.^49^ Such G4 formation has been associated with transcriptional stalling *in vivo*, a phenomenon exacerbated in CSB-deficient cells. These observations suggest that CSB is required to resolve G4 structures, which might be perceived as DNA damage, to restore transcriptional activity. Interestingly, subsequent investigations by our group revealed that CSB only exhibits resolvase activity toward rDNA G4s when folded following a multimolecular stoichiometry.^1^ We have subsequently demonstrated that CSB preferentially binds to any mG4s with astonishing picomolar affinity (Figure 4b,c).^1^

One noteworthy aspect is that the nucleolus, where the rDNA is stored and CSB mainly localizes (Figure 4d,e), is a well-known membrane-less organelle that arises from biomolecular condensation.^50^ In fact, nucleoli are formed via LLPS, and their organization into a phase-separated structure is critical for their biological functions.^50^ Previous studies have shown that nucleolar assembly is driven by multivalent interactions between proteins and protein-nucleic acids.^51^ For instance, the protein nucleophosmin (NPM1) has been shown to associate with arginine-rich proteins and rRNA, promoting the condensation of nucleoli into a phase-separated structure.^52,53^ Considering these insights, our findings on CSB suggest that this protein may be involved in a previously unrecognized mechanism of nucleolar assembly, where it stabilizes mG4s, ultimately leading to the biocondensation of nucleoli. Therefore, it is plausible that a similar mechanism might be exploited in transcriptional regulation, for example, in the assembly of super-G4s. These hypotheses have yet to be tested with dedicated tools and experiments. Still, they might delineate a paradigm shift vision by which the structural nature of nucleic acids can actively contribute to the formation of biomolecular condensates in a cellular context.

In the context of transcriptional regulation, CSB could potentially recognize and bind to endogenous mG4s to facilitate the clustering of distal genomic *loci*, which may drive phase separation and transcriptional activation. Given CSB’s known chromatin remodeling activity, we speculate that this clustering could also induce a three-dimensional reorganization of the chromatin, which may also contribute to altered transcriptional activity. This would offer a new and compelling avenue for future research into the role of G4s in transcriptional regulation, expanding the functional role of G4s beyond the canonical unimolecular structures to the multimolecular structures that have often been overlooked and deemed biologically irrelevant.

## Conclusion and Outlook

The function of G4s as transcriptional regulators at gene promoters is becoming widely accepted but is often limited to local perturbations of the epigenetic landscape or transcription factor binding. Nevertheless, an increasing body of evidence suggests that the involvement of G4s in transcription is far more complex and potentially intertwined with other biological processes including three-dimensional chromatin organization and phase separation. In this Account, we described key findings from our group that contribute to this evolving paradigm, highlighting the critical functional role that multimolecular G4s established between distal genomic regions could potentially play in this context. Notably, we and others have observed that nonpromoter G4s, particularly those at enhancers, can also be heavily linked to transcriptional activation as much as G4s formed at gene promoters. Our study and recent literature also underline how a cluster of G4s (super-G4s) can act as superenhancers and may serve as highly potent regulators of transcriptional enhancement. Although the mechanism underlying super-G4 formation remains largely unknown, our research on (GGGGCC)_*n*_ repeat expansion offers potential insights. We demonstrated that these repeat expansion sequences can form multimolecular G4s (mG4s) that can cluster into phase-separated aggregates, suggesting that G4 clusters might initiate LLPS in the absence of proteins. This leads us to speculate that super-G4s may also form via such mechanisms and potentially through LLPS. Additionally, we have researched a protein called CSB, which displays a high affinity and selectivity to mG4s. Given that CSB is a chromatin-remodeler, we hypothesize that proteins of this nature might be recruited to, or even drive, the phase separation of super-G4s, subsequently altering chromatin structure to facilitate enhanced transcription.

While our studies and the current literature are still in their infancy and will require additional experimental validation, it is becoming increasingly evident that the formation of G4 structures, particularly long-range multimolecular G4s, could play a key role in chromatin organization that goes well beyond simple protein recruitment and local chromatin accessibility. Our research strongly indicates that the chemical and physical properties of networks generated by long-range mG4s can trigger phase separation and selectively recruit chromatin-remodeling proteins, which are established markers of transcriptional regulation. It is thus conceivable that mG4s play a specific functional role in orchestrating chromatin architecture in a much more complex way than initially anticipated.
